# Rotavirus infection induces ferroptosis through NCOA4-mediated ferritinophagy in IPEC-J2 cells

**DOI:** 10.1186/s40104-026-01457-0

**Published:** 2026-06-30

**Authors:** Ye Zhao, Xia Dong, Qingyuan Lan, Luqian She, Yongsheng Hu, Lei Chen, Mailin Gan, Lili Niu, Yan Wang, Xiaofeng Zhou, Linyuan Shen, Li Zhu

**Affiliations:** 1 State Key Laboratory of Swine and Poultry Breeding Industry, College of Animal Science and Technology, Sichuan Agricultural University, No. 211, Huimin Road, Wenjiang District, Chengdu City, 611130 China; 2 Key Laboratory of Livestock and Poultry Multi-Omics, Ministry of Agriculture and Rural Affairs, College of Animal Science and Technology, Sichuan Agricultural University, No. 211, Huimin Road, Wenjiang District, Chengdu City, 611130 China; 3 Farm Animal Genetic Resources Exploration and Innovation Key Laboratory of Sichuan Province, Sichuan Agricultural University, No. 211, Huimin Road, Wenjiang District, Chengdu City, 611130 China

**Keywords:** Ferritinophagy, Ferroptosis, IPEC-J2 cell, NCOA4, Rotavirus

## Abstract

**Background:**

Rotavirus (RV) infection damages mature intestinal epithelial cells and is a leading cause of severe diarrhea in infants and young animals. However, the host cell death pathways involved in RV infection remain incompletely understood. This study used an RV-infected IPEC-J2 cell model and integrated transcriptomic, proteomic, and functional assays to investigate whether RV infection is associated with ferroptosis and NCOA4-mediated ferritinophagy.

**Results:**

High-throughput sequencing and data-independent acquisition (DIA)-based quantitative proteomic analysis showed enrichment of ferroptosis, autophagy, and cell death-related signaling pathways after RV infection. Functional assays showed that RV infection was associated with intracellular Fe^2+^ accumulation and lipid peroxidation. The ferroptosis inducer (erastin) increased VP6 expression, whereas the ferroptosis inhibitor (Fer-1) attenuated this effect. NCOA4, a ferritinophagy receptor, was upregulated after RV infection. NCOA4 knockdown reduced RV-associated Fe^2+^ accumulation, ROS production, ferritin degradation-related changes, and VP6 expression, whereas NCOA4 overexpression enhanced these responses. The autophagy inhibitor (3-MA) and the lysosomal blocker (CQ) attenuated RV-associated ferroptosis markers and VP6 expression, consistent with involvement of the autophagy-lysosome pathway in ferritin turnover.

**Conclusions:**

These findings support a model in which RV infection promotes ferroptosis-associated changes and VP6 expression through NCOA4-mediated ferritinophagy in IPEC-J2 cells. As this study was performed in vitro and did not directly measure epithelial transport, barrier function, infectious viral yield, or diarrheal outcomes, further in vivo studies are needed to determine the contribution of this pathway to RV pathogenesis.

**Supplementary Information:**

The online version contains supplementary material available at 10.1186/s40104-026-01457-0.

## Introduction

Rotavirus (RV), a non-enveloped double-stranded RNA (dsRNA) virus, is an important pathogen that causes severe acute gastroenteritis in young animals [1, 2]. It is estimated that 1 in every 260 children born worldwide will die from RV before the age of 5 [3]. The prevalence of RV in piglets varies from 3.3% to 67.3% [4], and the prevalence of pig farms reaches 61%−74% [5, 6]. Although vaccination has significantly reduced the infection rate, the emergence of RV variants and the complexity of host defense mechanisms still pose challenges to disease prevention and control [7]. Previous studies found that RV has tropism for mature intestinal epithelial cells, leading to destruction of intestinal epithelial cell homeostasis and increased epithelial renewal [8–10]. At present, the mechanism by which RV infection contributes to host cell injury and death are not fully understood, and deeper analysis of these processes may help identify new therapeutic strategies.

Ferroptosis is a unique cell death mode driven by iron-dependent phospholipid peroxidation [11], which mainly involves changes in iron homeostasis and lipid peroxidation metabolism [12]. Circulating iron in the body enters the cell in the form of Fe^3+^ and binds to transferrin receptor 1 (TFR1) and is reduced to Fe^2+^. Part of the Fe^2+^ is stored in ferritin, which is composed of 24 subunits. Its subunit composition includes two functional components: heavy chain subtype (FTH1: ferritin heavy chain 1) and light chain subtype (FTL: ferritin light chain) [13]; the other part is released into the unstable iron pool in the cytoplasm [14]. Under external stimulation, the unstable iron pool can accumulate excessively and undergoes the Fenton reaction with hydrogen peroxide (H_2_O_2_) to produce hydroxyl and peroxyl free radicals, which will react with polyunsaturated fatty acids on the cell membrane to produce lipid ROS and lipid peroxidation, thereby promoting ferroptosis [15, 16]. Recent studies have shown that RNA viruses such as hepatitis C virus (HCV) [17] and coxsackievirus B3 (CVB3) [18] can induce ferroptosis by altering host iron metabolism pathways and may thereby create a cellular environment that favors viral replication. However, the relationship between RV infection and ferroptosis, as well as the role of ferroptosis in RV-host interactions, remains insufficiently defined.

Autophagy is a conserved intracellular catabolic process that transports cellular components to lysosomes for degradation [19]. Autophagy can contribute to ferroptosis through cargo-specific autophagy, including ferritinophagy [20, 21]. Ferritinophagy is a core link in the regulation of ferroptosis. Nuclear receptor coactivator 4 (NCOA4), as a specific receptor for ferritinophagy, directly recognizes and binds to FTH1, mediating the entry of ferritin (FTH1/FTL) into the autophagosome and its subsequent fusion with the lysosome. Autophagic degradation of ferritin releases Fe^2+^ and increases the intracellular free Fe^2+^ concentration [22–25]. Our previous study found that RV infection was associated with ferroptosis-related changes in cells [26]. At the same time, transcriptomic and proteomic analyses indicated enrichment of ferroptosis- and autophagy-related signaling pathways after RV infection, suggesting that RV may induce ferroptosis by regulating ferritinophagy. However, whether NCOA4 is involved in RV infection-mediated iron metabolism reprogramming and how this process occurs remain unclear.

Based on this background, this study constructed an RV-infected IPEC-J2 cell model and combined multi-omics analysis with functional validation experiments to investigate the molecular mechanism of RV infection-induced ferroptosis. We hypothesized that RV infection activates NCOA4-mediated ferritinophagy, promotes intracellular iron accumulation and lipid peroxidation, thereby contributes to ferroptosis-associated cellular injury and viral replication. This study may provide a new perspective for understanding the pathogenic mechanism of RV and lay a theoretical foundation for future evaluation of anti-RV strategies targeting ferroptosis.

## Materials and methods

### Rotavirus source and preparation

The OSU strain of RV (ATCC VR-893) was purchased from the Chinese Veterinary Drug Administration. It was passaged in MA104 clone 1 cells (ATCC#CRL-2378.1™). Virus propagation in IPEC-J2 cells was performed as described by Liu et al. [27]. First, RV was mixed with 5 μg/mL trypsin (Gibco, USA) and incubated in a 37 °C water bath for 30 min. Then, it was inoculated into IPEC-J2 cells and shaken every 15 min. After 60 min, the inoculum was removed and the cells were washed twice with phosphate buffered saline (PBS; Servicebio, China). Then, the cells was cultured by DMEM/F12 medium (Gibco, USA) until a pronounced cytopathic effect was observed. The infected material was collected, frozen and thawed three times, centrifuged (3,000 × *g*, 10 min), and the supernatant was collected and stored at −80 °C for later use.

### Determination of the median tissue culture infection dose (TCID_50_)

First, inoculate IPEC-J2 cells in a 96-well plate and cultured until they reached 100% confluence. Then, the RV stock solution was serially diluted with DMEM/F12 medium, and the cells were infected with the serially diluted RV in sequence. The cells were cultured in an incubator at 37 °C and 5% CO_2_, and the number of wells with and without cytopathic effect (CPE) was observed and recorded at each gradient. The TCID_50_ of the virus was calculated according to the formula of Reed-Muench method [28]. TCID_50_ was converted to plaque forming units (PFU), and the required amount of virus was determined based on the number of cells and the multiplicity of infection (MOI).

### Cell culture and virus infection

The IPEC-J2 cell line was isolated from the mid-jejunal epithelium of newborn unsuckled piglets and donated by Professor Per Torp Sangild of the University of Copenhagen, Denmark. IPEC-J2 cells were cultured in DMEM/F12 medium supplemented with 10% fetal bovine serum (ExCell Bio, China) and 1% penicillin–streptomycin (Beyotime, China) at 37 °C in a humidified incubator containing 5% CO_2_. During routine cell maintenance before RV infection, the culture medium was replaced every 24 h to provide fresh nutrients, maintain a relatively stable pH, reduce the accumulation of metabolic waste, and ensure that cells reached a healthy and uniform state before infection. IPEC-J2 cells were inoculated with pre-activated RV when cell confluence reached approximately 70%–80%. After 60 min of incubation at 37 °C, the non-bound virus fraction was carefully removed by washing with PBS, and fresh medium was then added for subsequent experiments. Unless otherwise specified, the medium was not repeatedly replaced during the RV infection period, in order to avoid disturbing viral infection and the concentrations of experimental treatments.

IPEC-J2 cells were seeded in 6-cm dishes at 0.8 × 10^6^ cells/mL, with 2 mL cell suspension per dish. IPEC-J2 cells were seeded in 6-well plates at a density of 0.3 × 10^6^ cells/mL, with 2 mL cell suspension added to each well. IPEC-J2 cells were seeded in 12-well plates at 0.1 × 10^6^ cells/mL, with 1 mL cell suspension per well. IPEC-J2 cells were seeded in 96-well plates at 0.01 × 10^6^ cells/mL, with 100 μL cell suspension per well. All treatment and control groups within the same experiment were seeded at the same density and cultured under identical conditions to reduce variation caused by cell number or confluence.

### Transcriptomics sequencing and analysis

IPEC-J2 cells were treated with or without 10 MOI RV for 24 h according to our previous study [26]. Then, the mRNA in cells was extracted using the RNAiso kit (TaKaRa, Japan). Sequencing was performed by Novogene Technology Co., Ltd. (Beijing, China). Fastp (version 0.23.1) was then used to perform basic statistics on the quality of the raw reads, including adapter contamination, low-quality nucleotides, and unrecognizable nucleotides. The clean reads were aligned to the porcine reference genome *Sus scrofa* 11.1 obtained from Ensembl. Finally, Microbiotics (https://www.bioinformatics.com.cn) was used for differential expression analysis, with the thresholds: *P* value < 0.05, |fold change| ≥ 2.

### Proteomic sequencing and analysis

IPEC-J2 cells were treated with or without 10 MOI RV for 24 h. Proteins from cells were extracted using SDT lysis buffer (100 mmol/L Tris-HCl, pH 8.0, 4% SDS, 100 mmol/L DTT). Protein digestion was performed using the FASP method. Purified peptides were then collected from cell samples for further liquid chromatography-mass spectrometry analysis. Liquid chromatography tandem mass spectrometry (LC–MS/MS) was performed using an Orbitrap Astral mass spectrometer (ThermoFisher, USA) and a Vanquish Neo UHPLC system (ThermoFisher, USA). MS data were retrieved in the UniProtKB (Swiss-Prot) database. The sequences were then annotated using information extracted from UniProtKB/Swiss-Prot, Kyoto Encyclopedia of Genes and Genomes (KEGG), and Gene Ontology (GO). GO and KEGG enrichment analysis was performed using Fisher's exact test with FDR correction for multiple testing.

### siRNA and overexpression plasmid transfection

When the cell density reached 30%, 20 nmol/L NCOA4 siRNA or negative control siRNA (GenePharma, China) was transfected into IPEC-J2 cells using Hieff Trans™ Universal Transfection Reagent (Yeasen, China). The siRNA sequences are shown in Table 1. For plasmid transfection, after the IPEC-J2 cell density reached 50%, lipofectamine 3000 (Invitrogen, USA) was used to transfect the control plasmid or NCOA4 overexpression plasmid (Tsingke, China) following the manufacturers’ instructions.

**Table 1:** The siRNA sequences

Name	Sequence (5′→3′)
siCtrl	Sense: UUCUCCGAACGUGUCACGUTT
	Antisense: ACGUGACACGUUCGGAGAATT
siNCOA4-1	Sense: CCUGCAAUUUCUUCAAUAATT
	Antisense: UUAUUGAAGAAAUUGCAGGTT
siNCOA4-2	Sense: GCCAGUUCAAUUGUCUUAUTT
	Antisense: AUAAGACAAUUGAACUGGCTT
si-NCOA4-3	Sense: GGGCUGAACAGCAAAUUAATT
	Antisense: UUAAUUUGCUGUUCAGCCCTT

### Cell viability assay

After 24 h of experimental treatment, cell viability was detected using the CCK-8 kit (Beyotime, China). 10 μL of CCK-8 reagent was added to each well and incubated in a 37 °C incubator for 1 h. Subsequently, the OD_450_ value was measured using a microplate reader.

### Lipid peroxidation assays

To detect the intracellular lipid peroxidation level, 5 μmol/L C11-BODIPY 581/591 probe (ThermoFisher, USA) was incubated with cells at 37 °C for 30 min, followed by washing twice with PBS. Subsequently, a fluorescence microscope (Leica, Germany) was used to measure fluorescence images at excitation/emission wavelengths of 485/525 nm.

### Determination of intracellular ROS levels

The intracellular ROS levels were monitored using 6-carboxy-2′,7′-dichlorodihydrofluorescein diacetate (H_2_DCFDA) fluorescent probe (ThermoFisher, USA). The cells after the experimental treatment were washed once with PBS and incubated in basal medium containing 1 μmol/L H_2_DCFDA for 1 h in the dark (37 °C). Subsequently, the cells were washed twice with PBS and the cell fluorescence was observed using a fluorescence microscope as described above.

### Measurement of intracellular Fe^2+^ levels

FerroOrange (Dojindo, Japan) was used to detect intracellular Fe^2+^ levels. After the experimental treatment, the cells were washed once with PBS and treated with 1 μmol/L FerroOrange at 37 °C for 20 min. Fluorescence images were measured with an excitation/emission wavelength of 543/580 nm using a fluorescence microscope.

### Immunofluorescence assays

IPEC-J2 cells were fixed with 4% paraformaldehyde (Servicebio, China) for 15 min and then washed three times with PBS for 5 min each. The cells were permeabilized with 0.1% Triton X-100 (Beyotime, China) for 10 min at room temperature and then washed three times with PBS. After blocking with 5% goat serum for 90 min, the cells were incubated with primary antibodies (VP6, Cat. No. ab181695, Abcam, UK; NCOA4, Cat. No. sc-373739, Santa Cruz, USA; LC3, Cat. No. 381544, Zenbio, China) at 4 °C overnight and washed three times with PBS. The cells were then incubated with secondary antibodies (Cat. No. BA1127, Boster, China; Cat. No. BA1031, Boster, China; Cat. No. BA1126, Boster, China) at room temperature for 2 h and washed three times with PBS. Finally, the cells were stained with DAPI (Beyotime, China) for 10 min, and the cell fluorescence was observed using a fluorescence microscope as described above.

### Quantitative reverse transcription PCR (qRT-PCR)

According to the manufacturer's instructions, cells were lysed and mRNA was extracted using the RNAiso plus kit (TaKaRa, Japan). RNA concentration and quality were measured using a spectrophotometer (ThermoFisher, USA). mRNA was reverse transcribed into cDNA using the Hiscript III RT SuperMix Reverse Transcription Kit (Vazyme, China). qRT-PCR was performed using TB GreenTM Premix Ex TapTMII (Takara, Japan). The primer sequences used for qRT-PCR are shown in Table 2. The relative expression levels of the target gene and the internal reference β-actin (*ACTB*) gene were calculated based on the comparative Ct value (2^−ΔΔCt^) method [29].

**Table 2:** The primer sequences used for qRT-PCR

Gene name	Sequence (5′→3′)	TM, °C
*VP6*	QF: TCAGTTCGTCAGGAATATGC	53.5
	QR: CTTGAAGGTGAGTAGTTGGT	
*NCOA4*	QF: GTTGGAGCCTTGTCCTATG	60.0
	QR: CAGTTCTACCCAGACTCGCAC	
*ACTB*	QF: TCTGGCACCACACCTTCT	59.0
	QR: TGATCTGGGTCATCTTCTCAC	

### Western blot analysis

IPEC-J2 cell lysis buffer was prepared by adding 1% PMSF (Beyotime, China), 2% protease inhibitor (Beyotime, China) and 2% phosphatase inhibitor (Beyotime, China) to RIPA lysis buffer (Beyotime, China). The cells were lysed at 4 °C for 30 min, centrifuged at 12,000 × *g* for 15 min, and the supernatant containing the protein was collected. Protein concentration was determined using a BCA kit (Beyotime, China). Then, 5 × SDS loading buffer (Beyotime, China) was added at a ratio of 1:4 and mixed, and denatured at 98 °C for 10 min. Then, protein samples, after separation via polyacrylamide gel electrophoresis (PAGE), were transferred onto a polyvinylidene fluoride (PVDF) membranes, which were blocked with 5% skim milk powder (Beyotime, China), incubated with primary antibodies (VP6, Cat. No. ab181695, Abcam, UK; NCOA4, Cat. No. sc-373739, Santa Cruz, USA; FTH1, Cat. No. R23306, Zenbio, China; FTL, Cat. No. A18051, ABclonal, China; LC3, Cat. No. 381,544, Zenbio, China; TFRC, Cat. No. 66180-1-Ig, Proteintech, USA; FPN, Cat. No. R23306, Zenbio, China; HMOX1, Cat. No. R24541, Zenbio, China; β-ACTIN, Cat. No. 380,624, Zenbio, China) at 4 °C overnight, and then incubated with corresponding horseradish peroxidase (HRP)-conjugated secondary antibodies.

### Statistical analysis

SPSS 21.0 software (SPSS, USA) was used for statistical analysis. All data are presented as mean ± standard error of the mean (SEM). Two-tailed *t*-test was used for two-group comparisons, and one-way analysis of variance (ANOVA) followed by Duncan's post hoc test was used for multiple comparisons. Statistical significance was set at *P* < 0.05. Images were visualized using BeyoECL Moon (Beyotime, China). β-Actin was used as a normalized target, and grayscale analysis was performed using ImageJ software to determine the grayscale value of the protein band.

## Results

### Integrated transcriptomic and proteomic analyses suggest RV-induced remodeling of cell death-related pathways

**Fig. 1:**
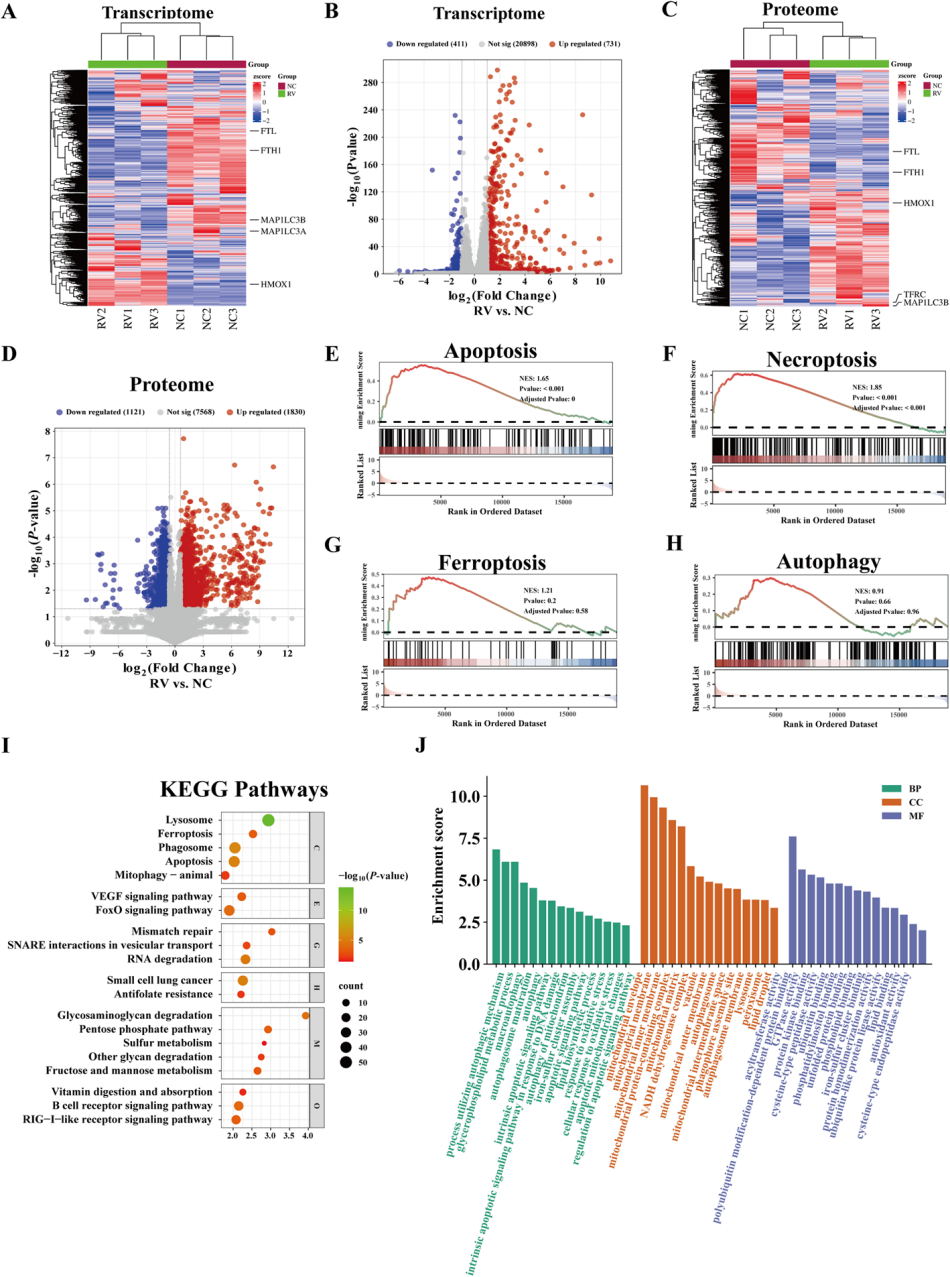
Transcriptomic and proteomic profiling of rotavirus (RV)-infected IPEC-J2 cells. After incubating IPEC-J2 cells with RV at 37 °C for 1 h, the unbound virus was removed by washing with PBS, and the cells were cultured in fresh medium for 24 h. Transcriptomic and proteomic analyses were performed to identify differentially expressed mRNAs and proteins between the RV-infected and control groups. **A** Heatmap showing the hierarchical clustering of differentially expressed mRNAs between the NC and RV groups. **B** Volcano plot showing differentially expressed mRNAs in RV-infected IPEC-J2 cells. **C** Heatmap showing the hierarchical clustering of differentially expressed proteins between the NC and RV groups. **D** Volcano plot showing differentially expressed proteins in RV-infected IPEC-J2 cells. **E**–**H** Gene set enrichment analysis (GSEA) showing the enrichment of apoptosis, necroptosis, ferroptosis, and autophagy-related pathways in RV-infected cells. **I** KEGG pathway enrichment analysis of differentially expressed proteins. **J** Gene Ontology (GO) enrichment analysis of differentially expressed proteins, including biological process (BP), cellular component (CC), and molecular function (MF) categories

To investigate molecular alterations induced by RV infection, IPEC-J2 cells were infected with RV at 10 MOI for 24 h and subjected to transcriptomic and proteomic analyses. The transcriptomic heatmap showed clear separation between the NC and RV groups, indicating that RV infection markedly altered mRNA expression profiles (Fig. 1A). Consistently, the volcano plot identified 1,142 differentially expressed mRNAs, including 731 upregulated and 411 downregulated mRNAs in RV-infected cells (Fig. 1B). Proteomic analysis also showed RV-associated molecular remodeling. The heatmap of differentially expressed proteins showed distinct clustering between the NC and RV groups (Fig. 1C). The proteomic volcano plot identified 2,951 differentially expressed proteins, including 1,830 upregulated and 1,121 downregulated proteins after RV infection (Fig. 1D). Gene set enrichment analysis showed that apoptosis and necroptosis pathways were positively enriched in RV-infected cells (Fig. 1E and F). Ferroptosis and autophagy pathways also showed positive enrichment patterns, suggesting their potential involvement in RV-induced cellular responses (Fig. 1G and H). KEGG pathway enrichment analysis of differentially expressed proteins revealed enrichment of pathways associated with lysosome, ferroptosis, phagosome, apoptosis, mitophagy, FoxO signaling, and RIG-I-like receptor signaling (Fig. 1I). Furthermore, GO enrichment analysis showed that the differentially expressed proteins were mainly associated with macroautophagy, autophagy, oxidative stress, apoptotic signaling, mitochondrial components, autophagosomes, lysosomes, and lipid-related molecular functions (Fig. 1J).

### RV infection induced ferroptosis in IPEC-J2 cells

**Fig. 2:**
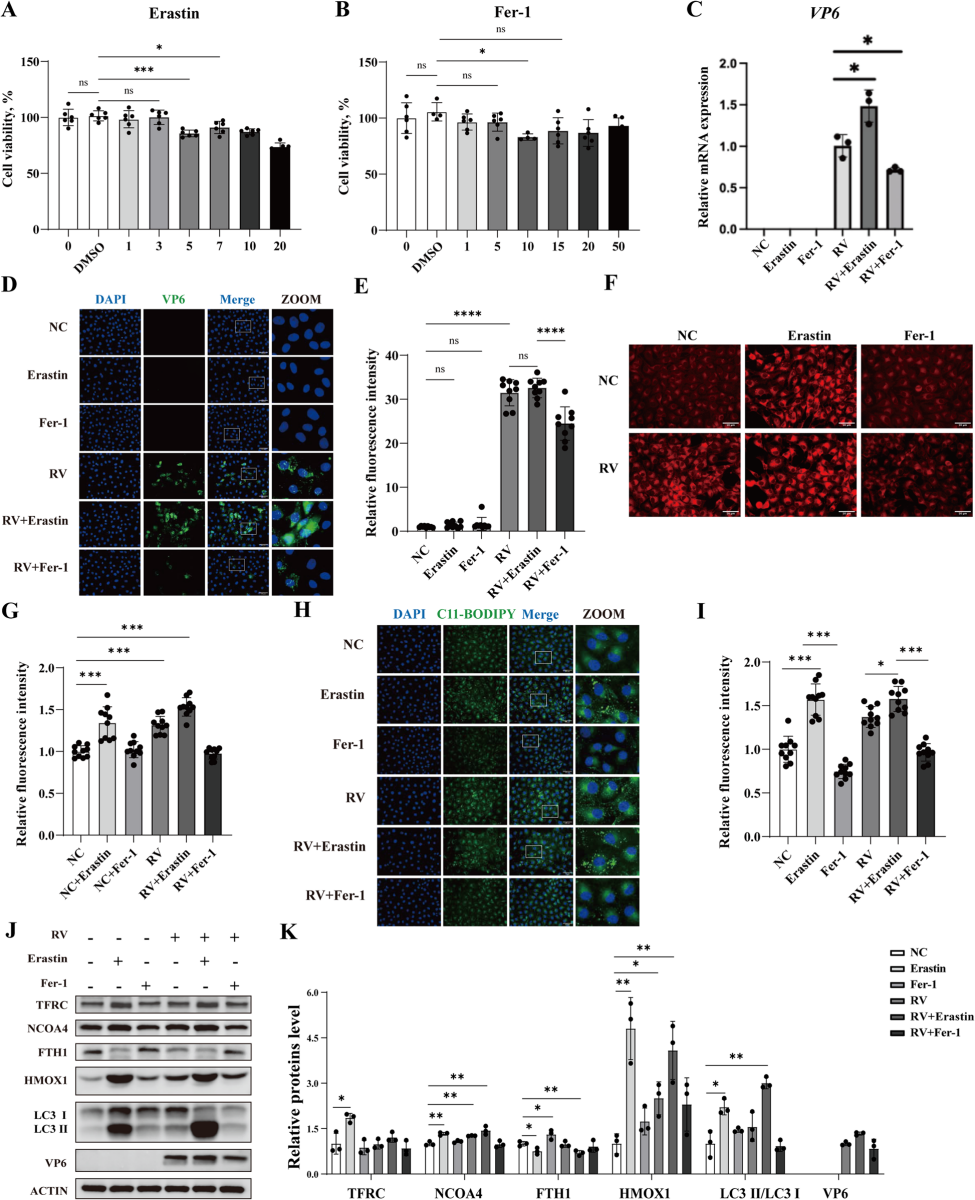
Rotavirus (RV) infection induces ferroptosis in IPEC-J2 cells. IPEC-J2 cells were cultured with RV for 1 h and then washed with PBS for unconjugated virus, followed by 24 h in a new medium containing 5 μmol/L erastin or 10 μmol/L Fer-1. **A** Cell viability of IPEC-J2 cells treated with different concentrations of erastin for 24 h. **B** Cell viability of IPEC-J2 cells treated with different concentrations of Fer-1 for 24 h. **C** Relative mRNA expression of *VP6* detected by qRT-PCR. **D** Representative immunofluorescence images of VP6 protein. **E** Quantification of VP6 fluorescence intensity. **F** Representative fluorescence images of intracellular Fe^2+^ levels detected using FerroOrange. **G** Quantification of relative Fe^2+^ fluorescence intensity. **H** Representative fluorescence images of lipid peroxidation detected using C11-BODIPY 581/591. **I** Quantification of relative C11-BODIPY fluorescence intensity. **J** Representative Western blot images of TFRC, NCOA4, FTH1, HMOX1, LC3-I/II, and VP6. **K** Quantification of relative protein levels. Data are presented as the mean ± SEM from at least three independent experiments. ^*^P < 0.05, ^**^P < 0.01, ^***^P < 0.001, ^****^P < 0.0001; ns, not significant. Scale bar = 50 μm

To explore the relationship between RV infection-induced IPEC-J2 cell damage and ferroptosis, we evaluated the toxic effects and optimal concentrations of ferroptosis inducers (erastin) and ferroptosis inhibitors (Fer-1) by detecting cell activity. As shown in Fig. 2A and B, 5 μmol/L erastin and 10 μmol/L Fer-1 were selected for subsequent experiments. To determine the effect of ferroptosis on RV replication, we detected RV component VP6. The qRT-PCR analysis revealed that, under RV infection conditions, erastin treatment significantly increased the *VP6* mRNA levels (Fig. 2C) and fluorescence intensity (Fig. 2D and E), and these effects were attenuated by Fer-1. FerroOrange is a fluorescent probe that specifically detects labile Fe^2+^. Compared with the control group, the RV infection group and the RV and erastin co-treatment group showed increased FerroOrange fluorescence intensity, whereas Fer-1 weakened the fluorescence signal (Fig. 2F and G). Because iron accumulation can promote ROS generation and lipid peroxidation, we used the C11-BODIPY 581/591 probe to measure intracellular lipid peroxidation. As shown in Fig. 2H and I, the RV infection group and the RV and erastin co-treatment group showed increased lipid peroxidation signals, and this effect was alleviated by Fer-1. The ratio of LC3II to LC3I was decreased by Fer-1, whereas HMOX1 protein expression increased after Fer-1 treatment. We also measured iron metabolism and lipid peroxidation-related proteins. Compared with the NC group, FTH1 protein levels were reduced and the TFRC, NCOA4, HMOX1, and LC3-II protein levels were increased in the RV-infected group and RV and erastin co-treatment group (Fig. 2J and K).

### NCOA4 mediated ferroptosis in RV-infected IPEC-J2 cells

**Fig. 3:**
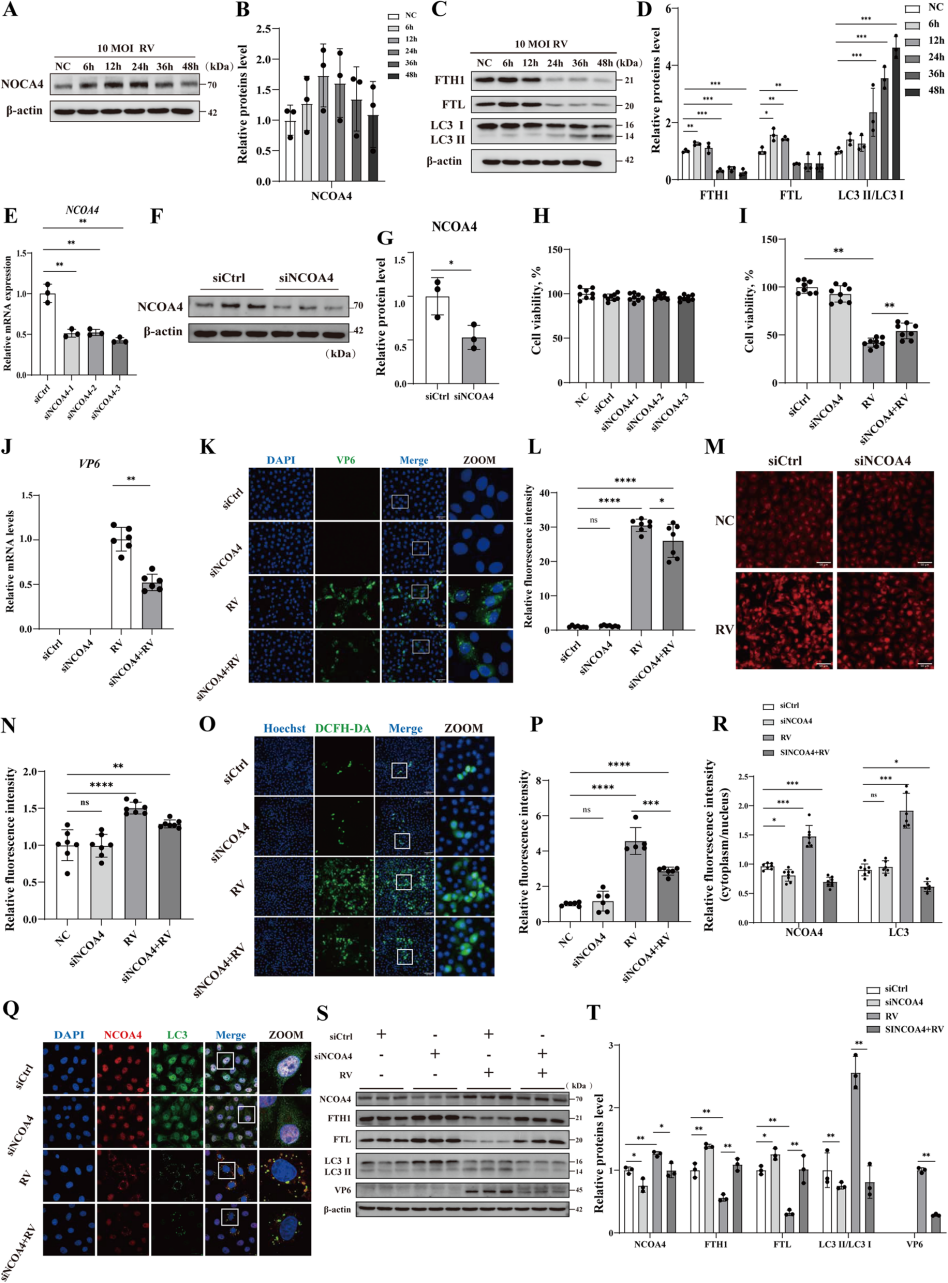
NCOA4 knockdown attenuates rotavirus (RV)-induced ferritinophagy and viral replication in IPEC-J2 cells. IPEC-J2 cells were pretreated with control siRNA or NCOA4 siRNA before being infected with RV at 10 MOI. Cells were co-incubated with RV at 37 °C for 1 h, washed with PBS to remove unbound virus, and then incubated for another 24 h in fresh medium. **A** and **B** NCOA4 protein expression and quantification at different time points after RV infection. **C** and **D** Representative Western blot images and quantification of FTH1, FTL, and the ratio of LC3-II to LC3-I at different time points after RV infection. **E** qRT-PCR analysis of *NCOA4* mRNA levels after transfection with three different NCOA4 siRNAs. **F** and **G** Western blot validation and quantification of NCOA4 knockdown efficiency. **H** Cell viability of IPEC-J2 cells after transfection with different NCOA4 siRNAs. **I** Effect of NCOA4 knockdown on cell viability in RV-infected IPEC-J2 cells. **J** Relative mRNA expression of *VP6* detected by qRT-PCR. **K** and **L** Representative immunofluorescence images and quantification of VP6 protein. **M** and **N** Representative FerroOrange fluorescence images and quantification of intracellular Fe^2+^ levels. **O** and **P** Representative DCFH-DA fluorescence images and quantification of intracellular ROS levels. **Q** and **R** Representative immunofluorescence images and quantification of NCOA4 and LC3 colocalization. **S** and **T** Representative Western blot images and quantification of NCOA4, FTH1, FTL, the ratio of LC3-II to LC3-I, and VP6 protein levels. Data are presented as the mean ± SEM from at least three independent experiments. ^*^P < 0.05, ^**^P < 0.01, ^***^P < 0.001, ^****^P < 0.0001; ns, not significant. Scale bar = 50 μm

To further explore the potential mechanism of ferroptosis induced by RV infection in IPEC-J2 cells, we performed siRNA-mediated knockdown of NCOA4 in IPEC-J2 cells. Western blot analysis revealed that RV infection can regulate NCOA4 and FTH1, FTL, and the ratio of LC3II to LC3I expression in a time-dependent manner (Fig. 3A–D). Three siRNA constructs were tested. Compared with control siRNA, NCOA4 siRNA significantly reduced NCOA4 mRNA and protein levels (Fig. 3E–G). Meanwhile, we evaluated the toxic effects of control siRNA and NCOA4 siRNA by detecting cell activity (Fig. 3H). Overall, siNCOA4-3 showed the highest knockdown efficiency and was selected for subsequent experiments. Compared with the RV-infected group, NCOA4 knockdown restored cell viability (Fig. 3I) and attenuated RV-induced increases in ROS production (Fig. 3O and P) and Fe^2+^ accumulation (Fig. 3M and N). In addition, NCOA4 siRNA decreased VP6 mRNA (Fig. 3J), protein levels (Fig. 3S and T) and fluorescence intensity (Fig. 3K and L) in RV-infected cells. As shown in Fig. 3Q and R, NCOA4 siRNA reduced the colocalization of NCOA4 (red) and LC3 (green) and caused LC3 to aggregate in a punctate manner. Western blot analysis showed that RV infection reduced FTH1 and FTL protein levels and increased the ratio of LC3II to LC3I, whereas NCOA4 siRNA partially attenuated the RV-induced reduction of FTH1 and FTL and decreased the RV-induced increase in the ratio of LC3II to LC3I (Fig. 3S and T).

### NCOA4-mediated ferroptosis promoted RV replication in IPEC-J2 cells

**Fig. 4:**
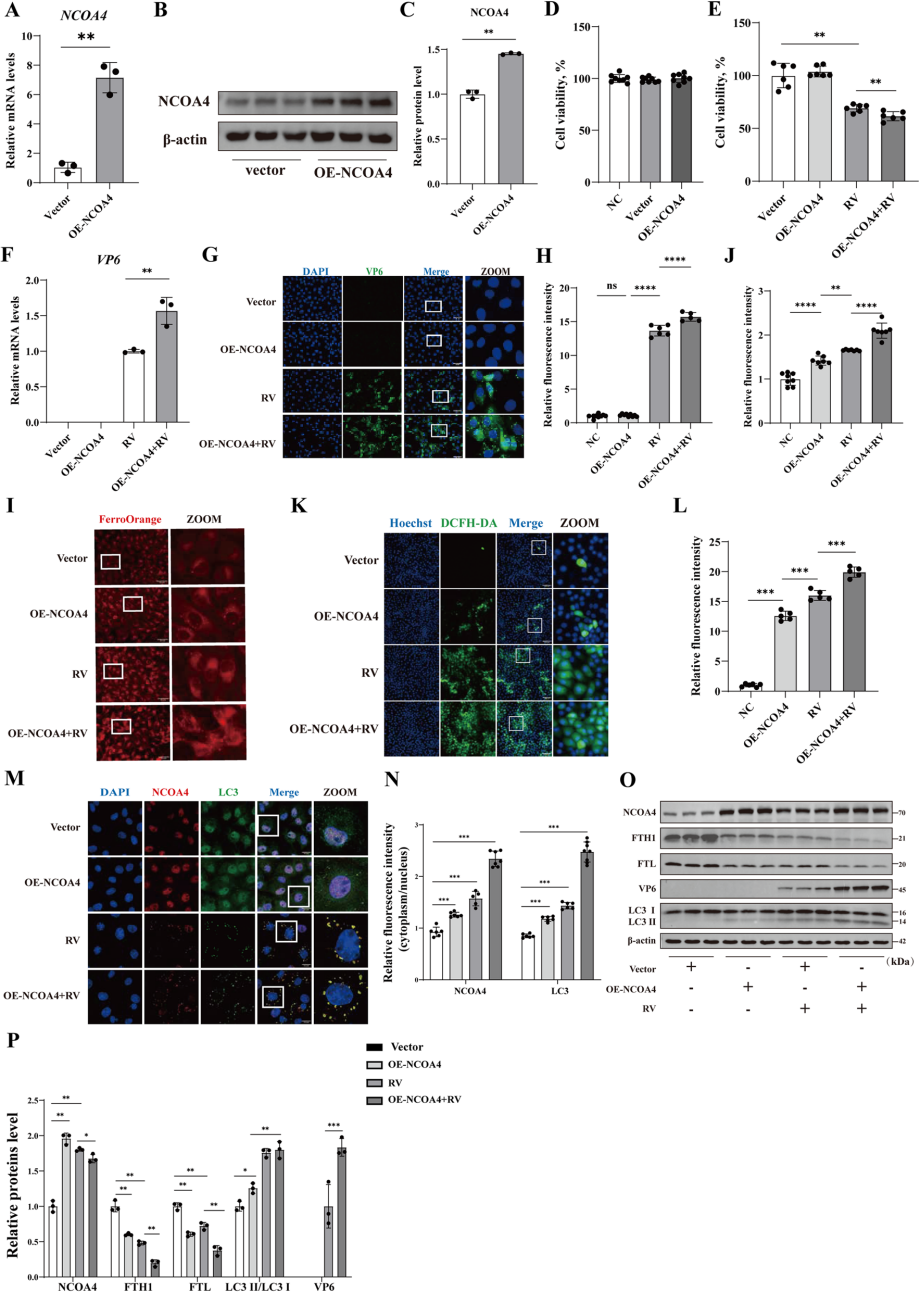
NCOA4 overexpression promotes ferroptosis and enhances rotavirus replication in IPEC-J2 cells. IPEC-J2 cells were pretreated with control vector or NCOA4-overexpression plasmid before being infected with RV at 10 MOI. Cells were co-incubated with RV at 37 °C for 1 h, washed with PBS to remove unbound virus, and then incubated for another 24 h in fresh medium. **A** Relative mRNA expression of *NCOA4* after NCOA4 overexpression. **B** and **C** Representative Western blot images and quantification of NCOA4 protein expression after NCOA4 overexpression. **D** Effect of NCOA4 overexpression on the viability of non-infected IPEC-J2 cells. **E** Effect of NCOA4 overexpression on cell viability in RV-infected IPEC-J2 cells. **F** Relative mRNA expression of *VP6* detected by qRT-PCR. **G** and **H** Representative immunofluorescence images and quantification of VP6 protein expression. **I** and **J** Representative FerroOrange fluorescence images and quantification of intracellular Fe^2+^ levels. **K** and **L** Representative DCFH-DA fluorescence images and quantification of intracellular ROS levels. **M** and **N** Representative immunofluorescence images and quantification showing the colocalization of NCOA4 and LC3. **O** and **P** Representative Western blot images and quantification of NCOA4, FTH1, FTL, VP6, LC3-I, and LC3-II protein levels. Data are presented as the mean ± SEM from at least three independent experiments. ^*^P < 0.05, ^**^P < 0.01, ^***^P < 0.001, ^****^P < 0.0001; ns, not significant. Scale bar = 50 μm

To further explore the role of NCOA4 in RV infection-induced ferroptosis, we transfected an NCOA4 overexpression plasmid into IPEC-J2 cells. Compared with the control group, NCOA4 overexpression significantly upregulated NCOA4 mRNA and protein levels (Fig. 4A–C). NCOA4 overexpression had no effect on cell viability in non-infected cells (Fig. 4D), but enhanced the RV-induced decrease in cell viability (Fig. 4E). Under RV infection conditions, NCOA4 overexpression significantly increased VP6 mRNA (Fig. 4F), protein levels (Fig. 4O and P), and fluorescence intensity (Fig. 4G and H), and increased Fe^2+^ accumulation (Fig. 4I and J) and ROS production (Fig. 4K and L). In addition, compared with the control group, immunofluorescence analysis showed that NCOA4 overexpression promoted the colocalization of NCOA4 (red) and LC3 (green) (Fig. 4M and N), and Western blot analysis showed that NCOA4 overexpression downregulated FTH1 and FTL proteins and upregulated LC3-II protein (Fig. 4O and P).

### NCOA4-mediated ferritinophagy promoted RV infection-induced ferroptosis in IPEC-J2 cells

**Fig. 5:**
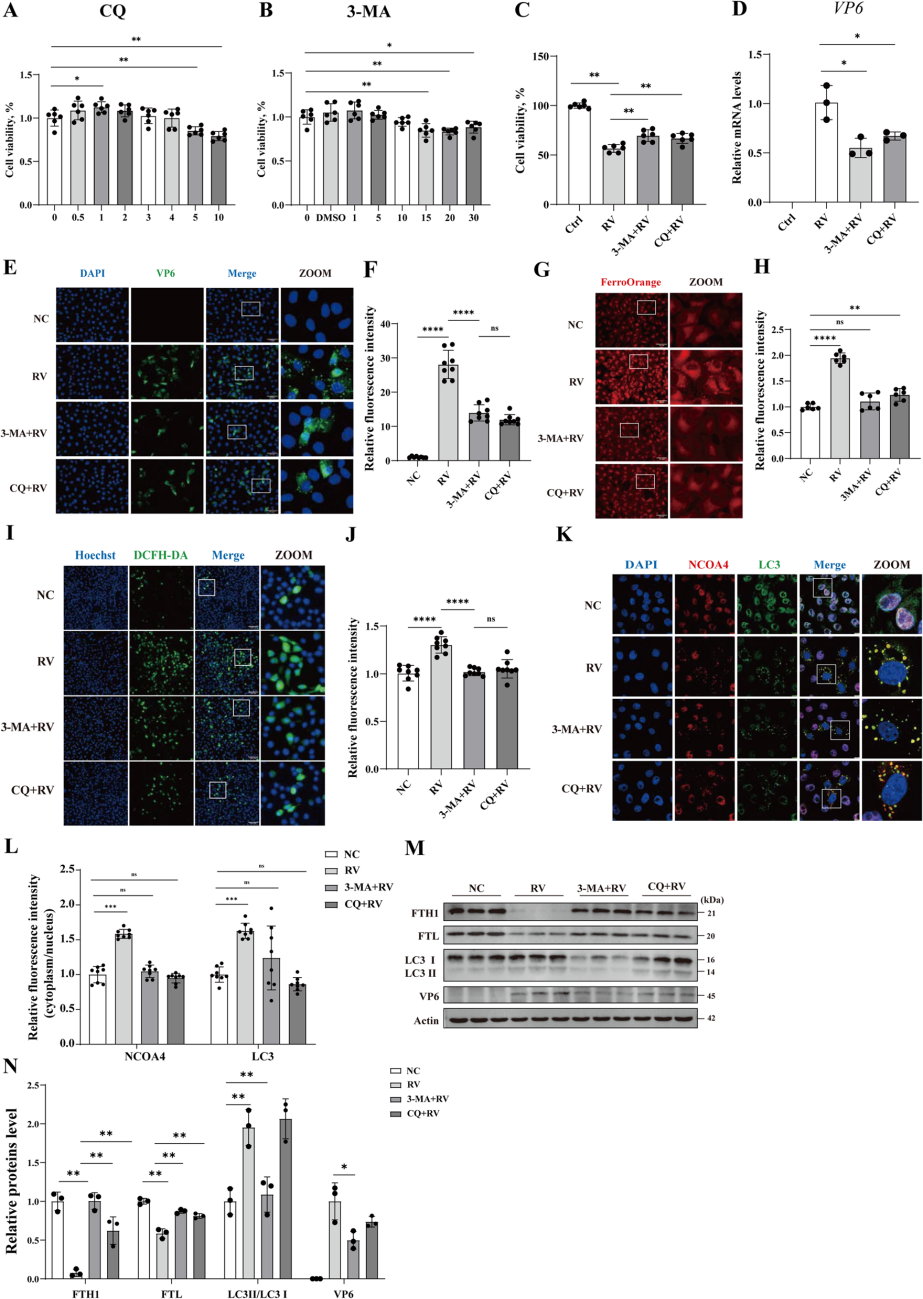
Inhibition of the autophagy–lysosome pathway attenuates RV-induced ferroptosis and viral replication in IPEC-J2 cells. IPEC-J2 cells were pretreated with 3-methyladenine (3-MA) or chloroquine (CQ) before being infected with RV at 10 MOI. Cells were co-incubated with RV at 37 °C for 1 h, washed with PBS to remove unbound virus, and then incubated for another 24 h in fresh medium containing the corresponding compounds. **A** and **B** Cell viability of IPEC-J2 cells treated with different concentrations of CQ or 3-MA. **C** Effects of 3-MA and CQ treatment on cell viability in RV-infected IPEC-J2 cells. **D** Relative mRNA expression of *VP6* detected by qRT-PCR. **E** and **F** Representative immunofluorescence images and quantification of VP6 protein expression. **G** and **H** Representative FerroOrange fluorescence images and quantification of intracellular Fe^2+^ levels. **I** and **J** Representative DCFH-DA fluorescence images and quantification of intracellular ROS levels. **K** and **L** Representative immunofluorescence images and quantification of NCOA4 and LC3 fluorescence signals. **M** and **N** Representative Western blot images and quantification of FTH1, FTL, LC3-II, and VP6 protein levels. Data are presented as the mean ± SEM from at least three independent experiments. ^*^P < 0.05, ^**^P < 0.01, ^****^P < 0.0001; ns, not significant. Scale bar = 50 μm

To further explore the underlying mechanism of ferroptosis in IPEC-J2 cells induced by RV infection, we investigated the cellular autophagy network. Based on the CCK-8 assay and Western blot analysis, we selected 4 mmol/L 3-methyladenine (3-MA, an autophagy inhibitor) (Fig. 5A) and 10 μmol/L chloroquine (CQ, a lysosome inhibitor) to assess ferritinophagy-related markers and targets (Fig. 5B). In RV-infected cells, 3-MA or CQ significantly improved cell viability (Fig. 5C) and led to marked reductions in VP6 mRNA (Fig. 5D), protein expressions (Fig. 5M and N), and fluorescence intensity (Fig. 5E and F). Compared with the RV infection group, 3-MA and CQ attenuated intracellular ROS production (Fig. 5I and J) and Fe^2+^ accumulation (Fig. 5G and H) in RV-infected IPEC-J2 cells. Notably, 3-MA or CQ treatment reduced the intracellular colocalization of NCOA4 (red) and LC3 (green) (Fig. 5K and L), upregulated FTH1 and FTL protein expression in RV-infected cells, and altered RV-induced LC3-II accumulation (Fig. 5M and N).

## Discussion

**Fig. 6:**
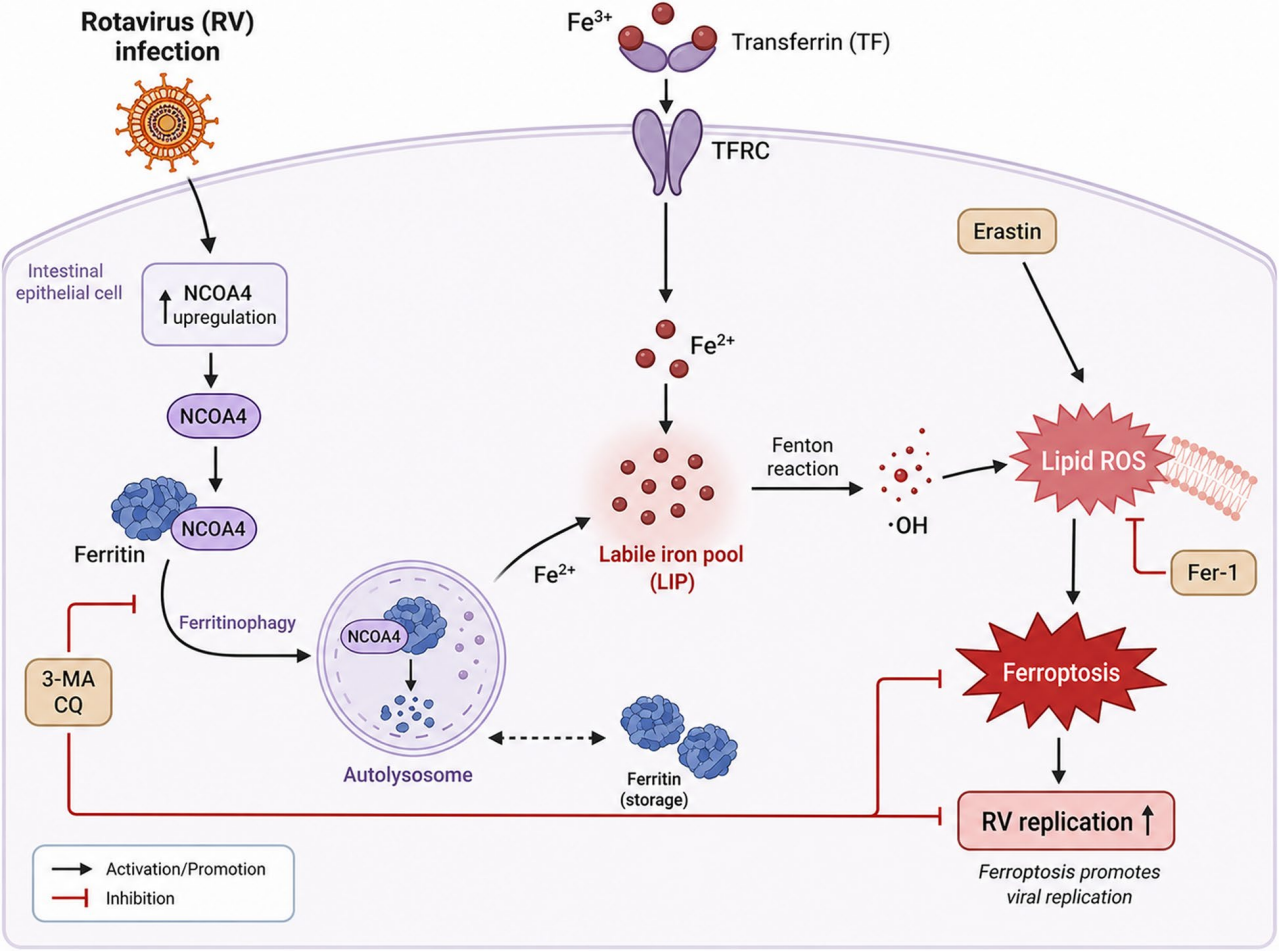
Diagram depicting the mechanism of RV induced ferroptosis in IPEC-J2 cells by regulating NCOA4-mediated ferritinophagy

The intestinal epithelium provides an important defense barrier against the entry of pathogens in the gut lumen. As a well-characterized intestinal epithelial cell line derived from the jejunum of a piglet, IPEC-J2 cell line has been widely used to characterize interactions between enterocytes and RV in vitro [28]. RV is an important cause of viral diarrhea in piglets and predominantly invades epithelial cells in the proximal intestine, causing villous atrophy and crypt hyperplasia. RV infection is also associated with severe watery diarrhea, dehydration and death in human and animals. Our previous studies demonstrated that RV infection can contribute to intestinal epithelial barrier damage and apoptosis, which may be involved in RV induced diarrhea in animals [26, 30, 31]. In recent years, ferroptosis, a form of iron-dependent cell death, has gradually attracted attention for its role in viral infection [18, 32, 33]. Ferroptosis regulation involves multiple pathways, including iron metabolism, oxidative stress, and autophagy [34, 35]. The present study provides evidence that RV infection is associated with ferroptosis-related changes in IPEC-J2 cells and suggests that NCOA4-mediated ferritinophagy contributes to VP6 expression and cell injury in this model (Fig. 6).

### RV infection induced ferroptosis in IPEC-J2 cells by regulating iron metabolism and lipid peroxidation

In the present study, transcriptomic and proteomic analyses showed enrichment of ferroptosis-related signaling pathways after RV infection. The main feature of ferroptosis is iron-dependent cell death mediated by lipid peroxidation of cell membranes [36]. By performing functional analysis in RV infected IPEC-J2 cells, we found RV infection increased intracellular Fe^2+^ accumulation and lipid peroxidation levels. Similar results have been reported in hepatocytes and Kupffer cells infected by the RNA virus HCV [37]. A variety of viruses can disrupt redox homeostasis and promote lipid peroxidation cascades by regulating host-cell iron metabolism pathway, thereby supporting viral replication and spread [38]. It this study, erastin enhanced VP6 expression, whereas Fer-1 attenuated this effect, suggesting that ferroptosis-related changes may create a cellular environment that favors RV replication in IPEC-J2 cells. This result was consistent with previous studies indicating that CVB3 [18], swine influenza virus (SIV) [32], Newcastle disease virus (NDV) [33], and HCV [17] promoted viral replication through ferroptosis-related mechanisms. Iron uptake mediated by transferrin receptor (TFRC) provides raw materials for heme synthesis, and heme is a substrate for heme oxygenase-1 (HMOX1) [39]. When intracellular iron is insufficient, heme synthesis decreases, which may reduce the substrate availability of HMOX1; conversely, when iron is excessive, heme decomposition is enhanced and HMOX1 activity increases [40, 41]. Our study showed that RV infection promoted the upregulation of iron metabolism-related proteins TFRC and HMOX1, suggesting that RV infection may contribute to intracellular iron accumulation by promoting iron uptake and heme degradation. Similar mechanisms have also been reported in herpes simplex virus (HSV-1) [42] and human immunodeficiency virus (HIV) [43]. Collectively, these findings suggest that ferroptosis-related metabolic changes may be one strategy by which some viruses exploit host metabolism for efficient replication. It should be noted that HMOX1 and LC3-II changes alone are not sufficient to define ferroptosis. HMOX1 may have dual roles depending on the cellular context, acting either as a contributor to iron release or as part of an antioxidant stress response. Similarly, LC3-II accumulation alone cannot distinguish enhanced autophagosome formation from impaired autophagic degradation. Therefore, the conclusion that RV infection is associated with ferroptosis is based mainly on the combined evidence of Fe^2+^ accumulation, lipid peroxidation, altered ferritin/iron metabolism, and the rescue effect of Fer-1, rather than on HMOX1 or LC3-II expression alone.

### RV infection may induce ferroptosis through NCOA4-mediated ferritinophagy

Ferroptosis is often accompanied by ferritinophagy [44]. In ferritinophagy, NCOA4 mediates autophagic degradation of ferritin (FTH1/FTL), resulting in Fe^2+^ release [22, 24]. Our research found that NCOA4 protein increased in a time-dependent manner after RV infection. NCOA4 knockdown attenuated Fe^2+^ accumulation, ROS production, ferritin degradation-related changes, and VP6 expression, while NCOA4 overexpression enhanced these responses. A similar result was reported in human parainfluenza virus 2 (hPIV2) [45]. These results suggest that NCOA4 contributes to RV infection-associated ferroptosis-related changes. In terms of transport mechanism, the NCOA4-ferritin complex interacts with the autophagy marker protein microtubule-associated protein 1 lightchain 3 (LC3, there are two forms of LC3-I and LC3-II) to achieve directional transport to the autophagosome, promote ferritin degradation and iron release, and complete the ferritinophagy process [46–48]. Our results showed that NCOA4-LC3 colocalization was accompanied by ferritin degradation-related changes, supporting a model in which RV infection activates NCOA4-mediated ferritinophagy and thereby increases free iron availability in IPEC-J2 cells. This mechanism differs from the restrictive effect of traditional autophagy on some viruses [49, 50] and suggests that RV may use selected autophagy-related pathways to support replication. However, additional studies, including NCOA4 rescue experiments and direct autophagic flux assays, are needed to confirm the specificity and directionality of this mechanism.

### RV infection may induce ferroptosis by regulating the autophagy-lysosome pathway

During autophagy, the formation of autophagosomes and double-membrane vesicles is essential [51]. Then, the autophagosome fuses with the lysosome, the membrane compartment containing the cargo is cleaved, and the contents are degraded in the cell [51, 52]. Previous studies demonstrated that viruses can hijack autophagosomes to provide metabolites and energy for self-replication [53, 54]. Our results showed that 3-MA and CQ significantly reversed the decrease in cell viability, ROS accumulation, and Fe^2+^ release caused by RV infection downregulated VP6 expression. These results indicated that autophagosomes formation and lysosomal function contribute to RV infection-associated ferritinophagy in IPEC-J2 cells. During autophagy, the LC3-I precursor is processed into LC3-II and combined with phosphatidylethanolamine (PE) to attach to the autophagosome membrane [55]. Therefore, LC3-II is generally considered a marker of the autophagosome membrane and is often used to detect and track the occurrence and progression of autophagy [56, 57]. It is worth noting that 3-MA and CQ alleviated the RV infection-induced reduction of ferritin (FTH1, FTL) expression and inhibited LC3-II accumulation, suggesting that RV may promote ferritin degradation and free iron release by regulating the autophagy-lysosome pathway in IPEC-J2 cells. These findings support involvement of the autophagy-lysosome pathway in RV-associated ferroptosis-related changes and provide a theoretical basis for evaluating whether this pathway can be targeted to modulate RV infection.

Although our study revealed that RV infection induces NCOA4-mediated ferritinophagy and ferroptosis-related changes in IPEC-J2 cells, these in vitro findings cannot fully explain the diarrheal phenotype of RV infection. RV-induced diarrhea is multifactorial and involves villus enterocyte injury, malabsorption, altered electrolyte transport, NSP4-mediated secretion, and impaired epithelial barrier function [9, 58, 59]. In particular, RV and NSP4 have been reported to inhibit Na^+^-solute symport coupled with water transport, including SGLT1-mediated Na^+^-glucose absorption, and to promote intestinal fluid loss [59]. In this study, we did not directly measure Na^+^/water absorption, chloride secretion, transepithelial resistance, or tight-junction integrity. Therefore, we cannot conclude that ferroptosis directly causes reduced sodium/water absorption or diarrhea. Nevertheless, RV-induced Fe^2+^ accumulation, ROS production, lipid peroxidation, and decreased epithelial cell viability suggest that ferroptosis may aggravate epithelial injury. Because epithelial integrity is essential for absorption and barrier function, NCOA4-mediated ferroptosis may partly contribute to RV-associated malabsorption and barrier dysfunction. Further in vivo studies in RV-infected newborn piglets are needed to confirm this possibility.

## Conclusions

This study provides evidence that RV infection is associated with ferroptosis-related changes in IPEC-J2 cells and supports a model in which NCOA4-mediated ferritinophagy contributes to this process. The data indicate that the autophagy-lysosome pathway is involved in RV-associated ferritin turnover, iron accumulation, oxidative stress, and VP6 expression. However, because the study was conducted in an in vitro cell model and did not directly measure infectious viral yield, epithelial transport, barrier function, or in vivo diarrheal outcomes, the proposed "RV-NCOA4-ferritinophagy-ferroptosis" regulatory axis should be interpreted as a mechanistic model that requires further validation in animal models and primary intestinal systems.

## Supplementary Information

Additional file 1: Fig. S1. Uncropped PVDF membranes from Western blot analyses of RV-infected IPEC-J2 cells treated with erastin or Fer-1. Fig. S2. Uncropped PVDF membranes from Western blot analyses of RV-infected IPEC-J2 cells treated with NCOA4 siRNA. Fig. S3. Uncropped PVDF membranes from Western blot analyses of RV-infected IPEC-J2 cells subjected to NCOA4 overexpression. Fig. S4. Uncropped PVDF membranes from Western blot analyses of RV-infected IPEC-J2 cells treated with 3-MA or CQ.

## Data Availability

The datasets used and/or analysed during the current study are available from the corresponding author on reasonable request.

## Abbreviations

RV: Rotavirus
DIA: Data-independent acquisition
NCOA4: Nuclear receptor coactivator 4
dsRNA: Double-stranded RNA
TFR1: Transferrin receptor 1
FTH1: Ferritin heavy chain 1
FTL: Ferritin light chain
ROS: Reactive oxygen species
HCV: Hepatitis C virus
CVB3: Coxsackievirus B3
HMOX1: Heme oxygenase-1
TFRC: Transferrin receptor
LC3B: Light chain 3B
PE: Phosphatidylethanolamine
