# Supplementary material for: Rotavirus infection induces ferroptosis through NCOA4-mediated ferritinophagy in IPEC-J2 cells
Source: J Anim Sci Biotechnol. 2026 Jun 30;17:134. doi: 10.1186/s40104-026-01457-0 (PMC13317329; doi:10.1186/s40104-026-01457-0)
Supplement: Supplementary file 1 — Additional file 1: Fig. S1. Uncropped PVDF membranes from Western blot analyses of RV-infected IPEC-J2 cells treated with erastin or Fer-1. Fig. S2. Uncropped PVDF membranes from Western blot analyses of RV-infected IPEC-J2 cells treated with NCOA4 siRNA. Fig. S3. Uncropped PVDF membranes from Western blot analyses of RV-infected IPEC-J2 cells subjected to NCOA4 overexpression. Fig. S4. Uncropped PVDF membranes from Western blot analyses of RV-infected IPEC-J2 cells treated with 3-MA or CQ. [file 40104_2026_1457_MOESM1_ESM.docx]

**Fig. S1**


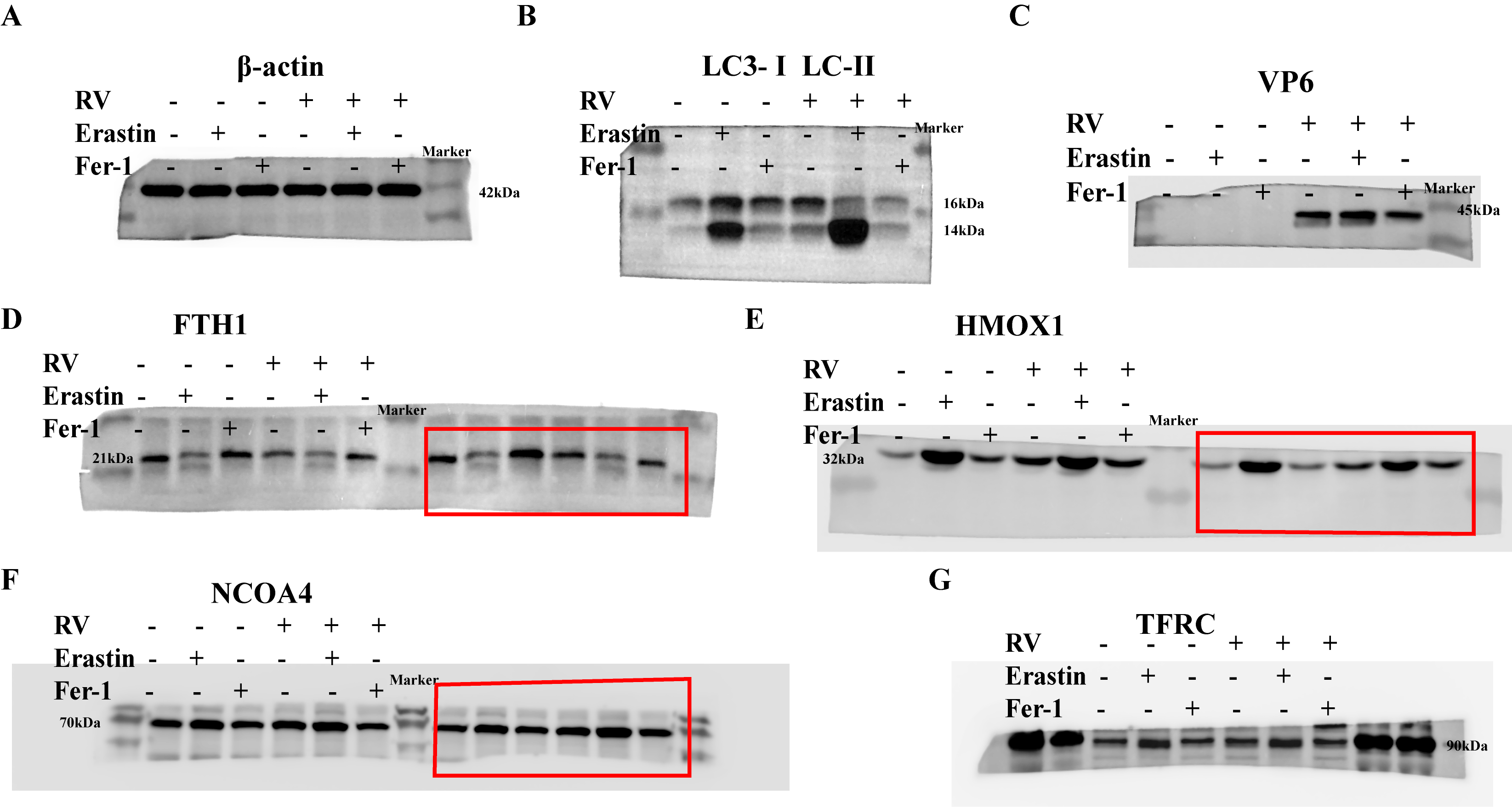


**Fig. S1** Uncropped PVDF membranes for Western blot analysis of RV infected IPEC-J2 cells treated with erastin or Fer-1. (A) β-actin, (B) LC3-I and LC3-II, (C) VP6, (D) FTH1, (E) HMOX1, (F) NCOA4, (G) TFRC. Red box indicated bands that were not included in this study.

**Fig. S2**

**Fig. S2** Uncropped PVDF membranes for Western blot analysis of RV infected IPEC-J2 cells treated with NCOA4 siRNA. (A) β-actin, (B) NCOA4, (C) β-actin, (D) FTL, (E) LC3-I and LC3-II, (F) FTH1, (G) NCOA4, (H) β-actin, (I) β-actin, (J) NCOA4, (K) VP6, (L) FTH1, (M) LC3-I and LC3-II, (N) FTL.

**Fig. S3**

**
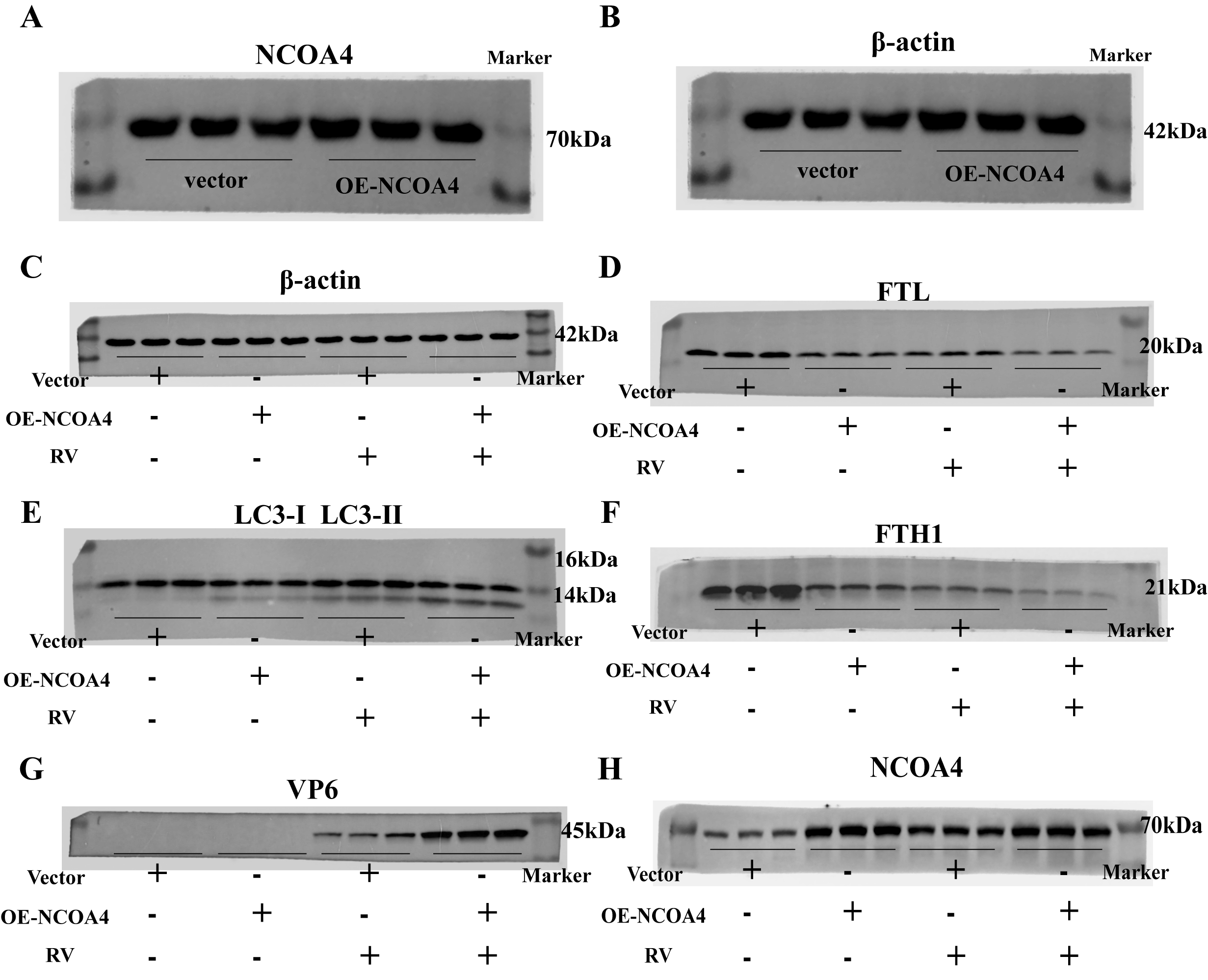
**

**Fig. S3** Uncropped PVDF membranes for Western blot analysis of RV infected IPEC-J2 cells treated with NCOA4 overexpression. (A) NCOA4, (B) β-actin, (C) β-actin, (D) FTL, (E) LC3-I and LC3-II, (F) FTH1, (G) VP6, (H) NCOA4.

**Fig. S4**

**
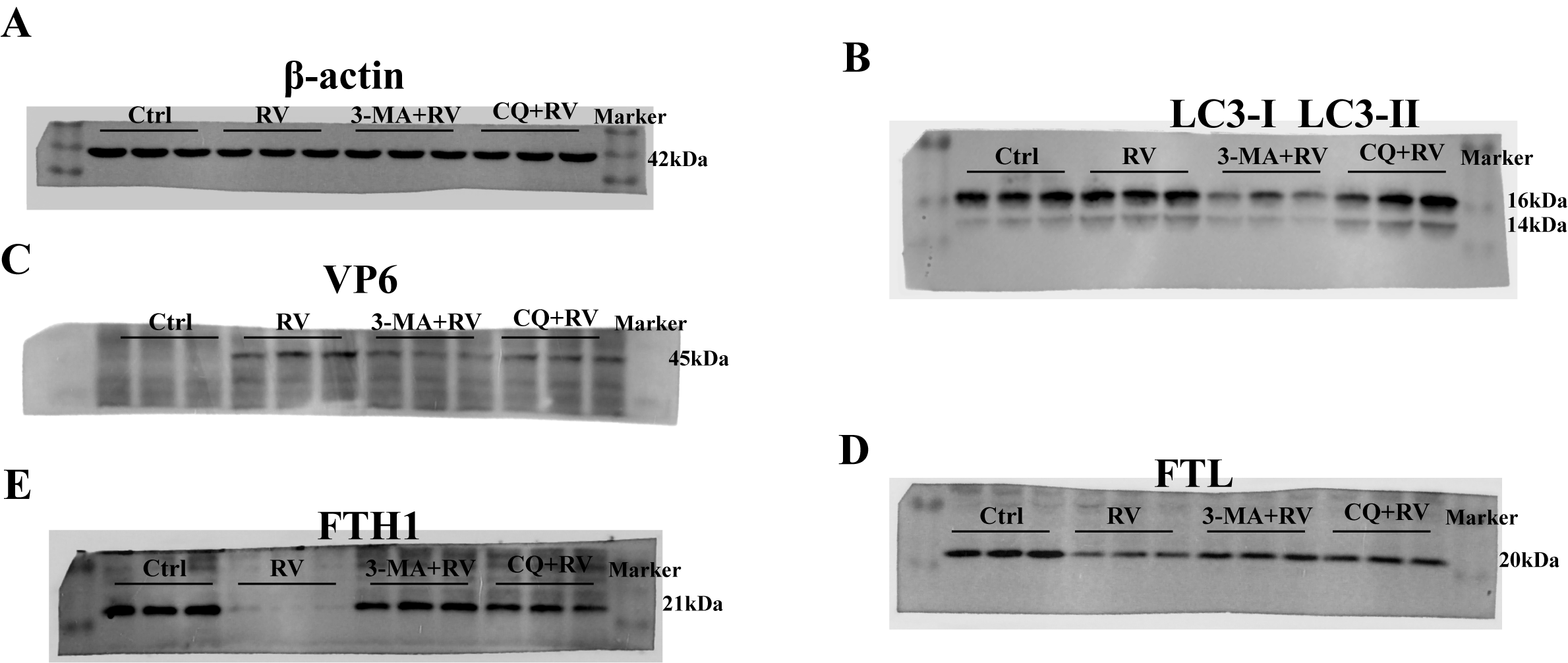
**

**Fig. S4** Uncropped PVDF membranes for Western blot analysis of RV infected IPEC-J2 cells treated with 3-MA or CQ. (A) β-actin, (B) LC3-I and LC3-II, (C) VP6, (D) FTL, (E) FTH1.
